# Multitasking During Simulated Car Driving: A Comparison of Young and Older Persons

**DOI:** 10.3389/fpsyg.2018.00910

**Published:** 2018-06-15

**Authors:** Konstantin Wechsler, Uwe Drescher, Christin Janouch, Mathias Haeger, Claudia Voelcker-Rehage, Otmar Bock

**Affiliations:** ^1^Institute of Physiology and Anatomy, German Sport University Cologne, Cologne, Germany; ^2^Faculty of Behavioral and Social Sciences, Institute of Human Movement Science and Health, Chemnitz University of Technology, Chemnitz, Germany

**Keywords:** task switching, dual-tasking, aging, cognitive-motor interference, ecological validity, virtual reality, car driving, multitasking

## Abstract

Human multitasking is typically studied by repeatedly presenting two tasks, either sequentially (task switch paradigms) or overlapping in time (dual-task paradigms). This is different from everyday life, which typically presents an ever-changing sequence of many different tasks. Realistic multitasking therefore requires an ongoing orchestration of task switching and dual-tasking. Here we investigate whether the age-related decay of multitasking, which has been documented with pure task-switch and pure dual-task paradigms, can also be quantified with a more realistic car driving paradigm. 63 young (20–30 years of age) and 61 older (65–75 years of age) participants were tested in an immersive driving simulator. They followed a car that occasionally slowed down and concurrently executed a mixed sequence of loading tasks that differed with respect to their sensory input modality, cognitive requirements and motor output channel. In two control conditions, the car-following or the loading task were administered alone. Older participants drove more slowly, more laterally and more variably than young ones, and this age difference was accentuated in the multitask-condition, particularly if the loading task took participants’ gaze and attention away from the road. In the latter case, 78% of older drivers veered off the road and 15% drove across the median. The corresponding values for young drivers were 40% and 0%, respectively. Our findings indicate that multitasking deteriorates in older age not only in typical laboratory paradigms, but also in paradigms that require orchestration of dual-tasking and task switching. They also indicate that older drivers are at a higher risk of causing an accident when they engage in a task that takes gaze and attention away from the road.

## Introduction

In everyday life, we often must perform multiple cognitive and motor tasks concurrently. For example, we steer a car along the road while watching for other traffic, responding to street signs and planning our route. As another example, we stroll on a sidewalk while avoiding obstacles, obeying traffic lights and chatting with another person. Experimental research about human multitasking began with a study by Jersild (1927), who reported that task performance deteriorates when two tasks are executed in an interleaved rather than in a blocked fashion. These performance decrements, later called “switching costs,” were attributed to the effort involved in disengaging from one task and adjusting to another task (Rogers and Monsell, 1995). In another line of research, two tasks were presented simultaneously or with a small stimulus onset asynchrony (Telford, 1931), which again led to performance decrements, called “dual-task costs.” The latter costs were attributed to a central processing bottleneck (Welford, 1952), task competition for a limited pool of attention (Kahneman, 1973) or competition for limited pools of specific processing resources (Wickens, 2002). These costs implicate a deterioration of performance, when the required attentional resources exceed the available ones. When participants have to handle very complex tasks or several tasks that require attention from the same pool, structural interferences impair the simultaneous handling of those tasks (Duncan et al., 1997). In real-life car driving, for example, a driver who passes a construction zone with narrow lanes must tightly control the car’s lateral position while at the same time keeping his distance to the preceding car. This forces the driver to direct his gaze at two spatially distinct locations concurrently, which is physically not possible, i.e., structural interference emerges (Heuer, 1996). In contrast, driving in narrow lanes without a leading car while listening to traffic announcements should lead to less structural interference, because the tasks don’t share sensory modalities.

Five decades of research provided indisputable evidence that abilities in cognitive (reviews in Craik, 1977; Verhaeghen et al., 2003) and motor-cognitive (Hahn et al., 2010; Beurskens and Bock, 2013) multitasking decline with advancing age. This age-related decline is not uniform, however. It affects mainly task combinations which draw heavily on working memory (Voelcker-Rehage et al., 2006; Voelcker-Rehage and Alberts, 2007; Chu et al., 2013) and/or visuo-spatial processing (Beurskens and Bock, 2012), and/or postural control (Boisgontier et al., 2013), and it emerges even if multitasking is limited to singular events such as an unexpected stimulus (Bock and Beurskens, 2011) or an unexpected error (Voelcker-Rehage et al., 2006). The decay of multitasking abilities in older age is also correlated with a decay of task-switching and memory-updating abilities (Kray and Lindenberger, 2000; Holtzer et al., 2005; Iersel et al., 2008; Liu-Ambrose et al., 2009), which suggests that it is at least partly due to an age-related impairment of executive functions.

It should be noted, that the age-related decline of multitasking abilities was observed in traditional laboratory paradigms and may not generalize unconditionally to real life. Laboratory research typically uses a limited number of well-defined stimuli (e.g., colored shapes on an otherwise blank screen), prescribes a limited number of elementary response alternatives (e.g., button presses) and associates those responses with no ecologically valid purpose. In contrast, everyday life offers an ever-changing flow of complex stimuli to which we respond by complex behavior in order to achieve a desirable goal. Furthermore, virtually all laboratory research was concerned with ‘multi’ tasking but actually presented only two tasks. This work therefore neglects the fact that in real life, we face an ever-changing sequence of concurrent tasks and must adjust to all of them in sequence. In other words, realistic multitasking incurs both dual-task costs and switching costs. Summing up, traditional laboratory paradigms suffer from behavioral impoverishment, lack of purpose and absence of the natural interplay between dual-tasking and task switching. The ecological validity (Chaytor and Schmitter-Edgecombe, 2003) of those paradigms may therefore be limited.

Several studies avoided behavioral impoverishment and lack of purpose by implementing realistic and immersive virtual-reality tasks such as car driving, street crossing or grocery shopping. Some of those studies dealt with dual-tasking: they combined virtual car driving or street crossing with a concurrent, cognitive or motor loading task. For example, simulated car driving has been combined with mobile texting (Drews et al., 2009), pattern detection or color memorizing (Cassavaugh and Kramer, 2009), and simulated street crossing with mobile internet use (Byington and Schwebel, 2013), listening to music or cellphone conversation (Neider and Kramer, 2011). The few studies which administered more than one concurrent task did so in separate blocks (Cassavaugh and Kramer, 2009; Neider and Kramer, 2011) and therefore still dealt with dual-tasking only; they didn’t address the natural interplay of dual-tasking and task switching encountered in everyday life. The present research goes beyond those studies by including such an interplay: our participants drove in a car driving simulator and concurrently performed not just one repetitive loading task, but rather an ever-changing sequence of loading tasks that involved different stimulus modalities, different cognitive processes and different output channels. To our knowledge, ours is the first study to introduce such a multitude of intermixed loading tasks.

Earlier virtual-reality studies reported a range of performance deficits under dual-task conditions. Thus, braking reaction times increased (Lamble et al., 1999; Lee et al., 2002; Strayer et al., 2003), gap estimations became less optimal (Brown et al., 1969), steering wheel control deteriorated (Kubose et al., 2006) and drivers responded to road hazards less often (Horberry et al., 2006). Findings were similar when loading tasks were administered while participants drove a real car on a closed-road circuit (Chaparro et al., 2005). The detrimental effects of loading tasks persisted even when drivers were encouraged to ignore them and to prioritize car braking (Levy and Pashler, 2008). Some of the available studies on dual-tasking in virtual reality dealt with older participants (Chaparro et al., 2005; Horberry et al., 2006; Anstey and Wood, 2011), but they didn’t sufficiently compare their performance to that of young persons. The effects of old age on realistic dual-tasking, let alone on the natural interplay of dual-tasking and task switching, are therefore still largely unknown. The main purpose of the present study was to close this gap in our knowledge.

It is well established that divided and selective attention deteriorate with advancing age (e.g., Rabbitt, 1965; McDowd and Shaw, 2000; review in Verhaeghen et al., 2003), especially when the tasks are complex (Zanto and Gazzaley, 2014) and that this downward trend is associated with poorer driving safety (Ball et al., 1993). It therefore is quite conceivable that the natural interplay of dual-tasking and task switching in realistic scenarios deteriorates as well. However, it has also been shown that age-related deficits observed in the laboratory may be absent under more natural conditions (Bock and Beurskens, 2010; Verhaeghen et al., 2012), possibly because older persons capitalize on their lifelong experience (Salthouse, 1984; Neider and Kramer, 2011). We therefore hypothesized that both young and older persons will show multitasking deficits when driving, that these deficits will be more pronounced when the loading task requires substantial visual processing and thus introduces structural interference, and that the magnitude of those deficits will be only moderately higher in older compared to young persons because of lifelong experience.

Summing up, our study is the first to compare young and older participants’ driving skills when exposed to a natural interplay of dual-tasking and task switching.

## Materials and Methods

### Participants

Sixty-three young (age 20–30 years; *M* = 23.17, *SD* = 2.83, females = 40) and 61 older (age 65–75 years; *M* = 69.97, *SD* = 2.96, females = 22) adults were recruited via postings at public places, social media, contacts with local senior networks as well as the website of the German Sport University Cologne and the Chemnitz University of Technology. Inclusion criteria were:

- A driving history of at least one trip per week during the last 6 months (self-report)
- No experience in multitasking research or simulator driving by self-report
- Good physical and mental health by self-report
- No history of stroke or brain surgery and no red-green color blindness by self-report
- A physician’s health clearance based on an exercise ECG within the last 6 months
- Visual acuity better than 20/60 (as assessed by the Freiburg Vision Test “FrACT”, Version 3.9.0); although the minimum requirement for a drivers’ license is 20/40 in most jurisdictions, driving safety is not degraded with a visual acuity of 20/60 (Keeffe et al., 2002).

Those who met these criteria underwent screening tests to assure that they don’t suffer from: cognitive impairment (assessed by the Mini-Mental State Examination; cutoff: 27/30 points), language comprehension deficits (assessed by the “Freiburger Sprachverständlichkeitstest”; cutoff: 50% word recognition at best hearing level) or obesity (cutoff: BMI ≥ 30).

The Edinburgh Handedness Inventory (cf. Oldfield, 1971) was used to determine hand dominance. Five Participants were left-handed, all others were right-handed. One participant was ambidextrous but used the right hand for the typing task. Persons who usually wore contact lenses, prescription glasses or hearing aids did so as well while participating in our study.

Participants were informed about the possibility to experience simulator sickness, and about their right to interrupt or abort the session at any time. Among the recruited persons, six young ones dropped out without giving a reason, three older ones because of simulator sickness and an older one because of reasons unrelated to our study. Registrations therefore were completed, and data were analyzed, from 63 young adults and 61 older ones.

This study was carried out in accordance with the recommendations of the Ethics Commission of the German Sport University with written informed consent from all subjects. All subjects gave written informed consent in accordance with the Declaration of Helsinki. The protocol was approved by the Ethics Commission of the German Sport University. Participants received 15 € per session (60€ in total).

### Driving Task

**FIGURE 1:**
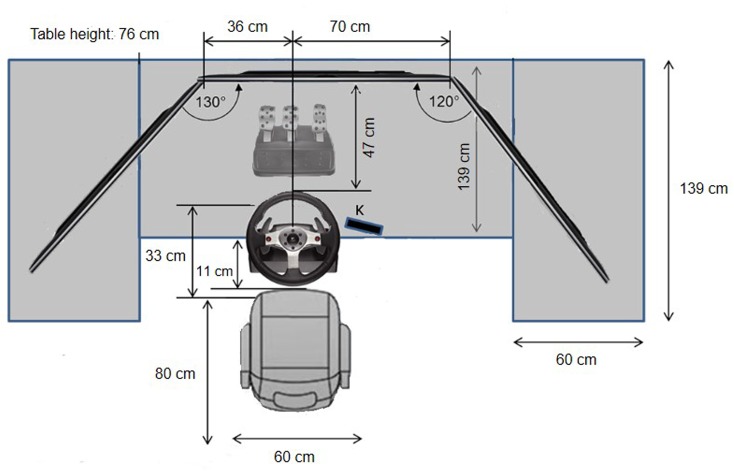
Driving setup, Top view. Keypad marked with ‘K,’ three monitors (black color in the picture) lined up as shown on top of three tables (gray color in the picture). Central table made transparent in the picture to show pedals. Seat, steering wheel and pedals lined up as shown.

**FIGURE 2:**
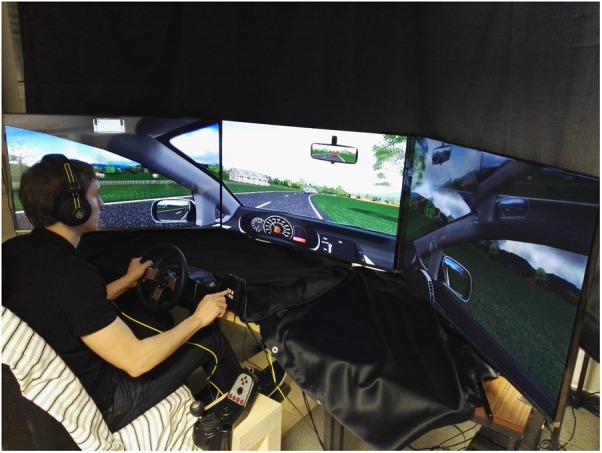
Driving simulator environment.

**Figure 1** shows a schematic top view of the setup, **Figure 2** shows a photo of the realization and the environment. Participants sat in a conventional car seat in front of three 48″ TV screens, which rendered the driver’s view of cockpit and surrounds with a total viewing angle of 195°. A steering wheel and pedal set (Logitech G27) were mounted in locations similar to a real car, and a numeric keypad (‘K’ in **Figure 1**) was mounted within easy reach. Participants wore a headset with microphone (shark zone H10, Sharkoon) not shown in **Figure 1**.

Commercially available driving simulator hard- and software (Carnetsoft^®^ version 8.0) was used to display a softly winding rural road without traffic lights or intersections. The driving environment was realistically portrayed with road signs, buildings and other vehicles (cars, busses, and trucks) which traveled in the opposing lane at constant speed. The landscape contained animals, trees, bushes, fences, straw bales, mountains and clouds in a blue sky. Participants drove a VW Golf with automatic transmission, and had full front and side view out of the cockpit. The dashboard displayed the typical devices including a speedometer. Two side-view and one rear-view mirrors were located in the usual locations, and presented the expected views.

Participants were instructed to follow a lead car which drove at a constant speed of 70 km/h. At irregular intervals, the lead car approached a construction site or a speed-restricted zone and slowed down to 40 km/h within 7 s. It kept this speed for 6 s, and then returned within 9 s to 70 km/h. Thus, participants had to slow down in order to avoid a collision, and to speed up afterwards in order to keep up with the leading car. We will refer to this maneuver as ‘braking task.’ Each driving trip was 25.7 km long, included 10 braking tasks and took about 25 min to drive.

When drivers didn’t keep up with the leading car and inter-vehicle distance exceeded 100 m, the leading car slowed down to 70% of the participants’ current speed until inter-vehicle distance decreased to 50 m, and then sped up again. This ensured comparable inter-vehicle distances for all participants and conditions.

### Loading Tasks

**FIGURE 3:**
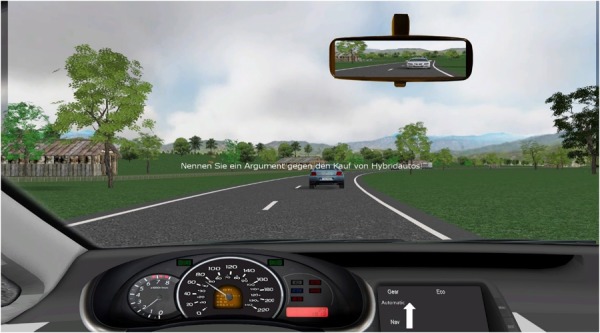
Screenshot out of the cockpit with visually displayed reasoning task.

A battery of loading tasks was presented in a mixed order, at unpredictable times. Task presentation was identical for every participant. Tasks were modeled after natural activities, involved different sensory modalities and required different types of responses. A given type of any task was not presented twice in succession via the same modality. The sound volume of auditory stimuli was individually adjusted for each participant. Each of the three following types of task was presented 20 times during a driving trip: 10 times visually for 5 s in the middle of the windshield and 10 times auditorily over headphones (Example in **Figure 3**).

**FIGURE 4:**
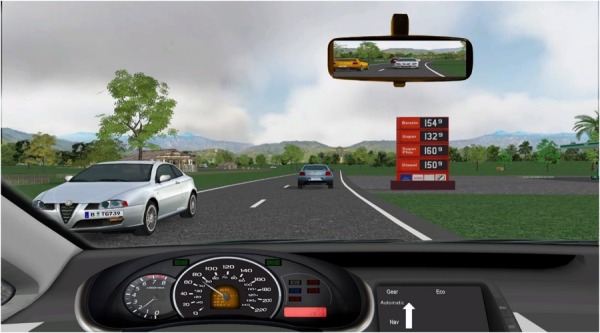
Screenshot out of the cockpit with visually displayed memory task.

- Typing: a three-digit number was presented, and participants responded by typing that number into the keypad. This task simulates operating, e.g., a radio receiver or GPS navigator.
- Reasoning: a question which couldn’t be answered by “yes” or “no” was presented, e.g., “What would be an argument against the taxation of sugar?” Participants responded verbally, and their response was registered by the headset microphone. This task simulates conversation with a car passenger or via a hands-free mobile phone.
- Memory: In the visual version, participants passed a gas station equally often appearing on the right or left side of the road and were asked over headphones whether the displayed price for premium gas was the same as at the preceding gas station immediately after (Example in **Figure 4**). In the auditory version, participants heard a traffic announcement over headphones and were then asked whether the reported congestion (highway number, location, length) was the same as in the preceding traffic announcement. In both task versions, participants respond verbally “yes” or “no” into the headset microphone.

### Procedures

Each participant completed four experimental sessions on separate days, with at least 1 day off in-between. This took between 8 and 28 days, depending on the participants’ availability. The first session included screening tests (to meet our inclusion criteria), driving simulator practice and practice of the loading tasks. Before the practice trials, participants received instructions and were encouraged to ask questions. Driving was practiced for 3–4 min, on the same course used for data collection. Loading tasks were practiced for 3–4 min on the same course as well, while the car drove in autopilot mode. The multitask condition (MT) was not practiced.

The subsequent three sessions were administered in an order that was balanced across participants. In one session, participants drove behind the leading car with no additional tasks (single-task driving, ST_D_). In another session, they drove behind the leading car while concurrently responding to the loading tasks (MT). In yet another session, the car drove in autopilot mode to provide a similar visual stimulation as in the other two sessions, and participants only responded to the loading tasks (ST_L_). The driving course was identical in all three conditions. Before the practice trials and at the beginning of the 2nd, 3rd, and 4th session, the examiner read aloud the pertinent instructions and explained every task separately. (S)he then withdrew from the participants’ view; during the remainder of the session, (s)he took notes and supervised the procedure without disturbing or interacting with the driver.

Participants also underwent cognitive and physical testing, and their street-crossing behavior was examined in a separate virtual-reality setup. This paper focuses on driving, a separate contribution in this issue deals with street crossing, and the other outcomes will be communicated later.

### Data Analysis

Driving performance in MT was analyzed within road segments of interest. Each segment started with the presentation of a loading task and ended 1 s before presentation of the next loading task. Segment duration varied, in dependence on driving speed and loading-task distance, in the range 17.46 ± 2.45 s (Mean duration ± standard deviation). We adopted this particular definition of road segments in order to analyze driving performance even when responses required substantial time for pondering and verbalizing. On rare occasions, reasoning took longer than the duration of the pertinent road segment; we then decided case by case whether the response was substantially completed and if not, marked it as ‘invalid.’

Since the driving course was identical in all three conditions, we could analyze participants’ performance in each condition within the same road segments (i.e., same road curvature and visual scenery). However, this similarity of the driving environment does not extend to the individual loading tasks: it is conceivable that on the average, one loading task was presented on curvier road segments and/or in a more cluttered visual scenery than another loading task. Differences between tasks are therefore confounded by differences between road conditions. By the same token, differences between modalities are confounded by differences between road conditions. Scattering of loading tasks along therefore added to the realism of our paradigm, but hinders comparisons between tasks and modalities.

The simulator software registered a range of continuous signals at a rate of 10 Hz. Among them were the lateral position of the driven car (0 m: car centered in its lane; <-0.78 m: right wheels off the road), and its distance from the lead car (0 m: bumpers touch). From these signals, we calculated the following parameters for each road segment of interest:

- Mean velocity
- Standard deviation of velocity (SD velocity)
- Mean lateral position
- Standard deviation of the lateral position (SD lateral position).

Furthermore, we calculated the following parameters for the typing and the memory task:

- Reaction time (RT): Interval between task presentation and response onset
- Correctness (COR): Proportion of all correct key presses in the typing task [0.00 (all wrong), 0.33 (one correct), 0.67 (two correct) or 1.00 (all correct), response correctness in the memory task (0 (wrong) or 1 (correct)].

Reaction time and COR in the typing task were determined by a software algorithm. RT in both other tasks was determined manually, by setting a cursor in the visually displayed voice tracks. COR in both other tasks was determined by listening to the voice tracks. We noticed during data analysis that in the memory task, older participants often started to respond even before the verbal question was completed. We therefore decided to exclude RT in the memory task from further analyses. All other parameters were averaged across the 10 repetitions of each task, excluding outliers as identified by the ± 3.29 SD criterion (Tabachnick et al., 2001).

### Statistical Analyses

Averaged scores were submitted to four-way analyses of variance (ANOVAs) with repeated measures on the factors Condition (ST and MT), Task (memory, reasoning, and typing) and Modality (visual and auditory) and the between-factor Group (young and older). We interpreted $\eta_{p}^{2}$ values < 0.06 as small, 0.06–0.14 as medium and >0.14 as large effects (Cohen, 1992). *P* < 0.05 was set for statistical significance. When the assumption of sphericity was violated in Mauchly’s tests, degrees of freedom were Greenhouse-Geisser corrected. We used IBM SPSS Statistics, version 25 (IBM Corp., Armonk, NY, United States) for those calculations.

## Results

### Driving Task

**FIGURE 5:**
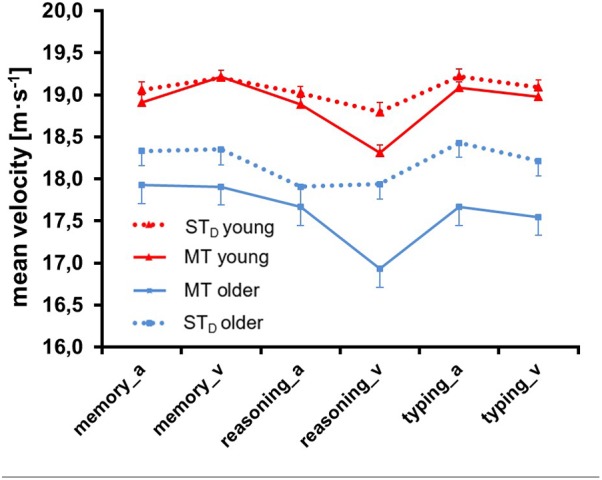
Mean velocity ± SE of both age groups in single task (ST_D_) and multitask (MT) conditions. Memory, reasoning and typing task were presented auditorily (_a) and visually (_v).

**Figure 5** illustrates the driving parameter *mean velocity* of both age groups in ST_D_ and in MT, separately for all six combinations of loading task and modality. ANOVA (see **Table 1**) yielded a significant main effect for Condition: participants drove more slowly in MT compared to ST_D_ (*F* = 12.07, *p* = 0.00, $\eta_{p}^{2}$ = 0.09, df = 1, 122). The mean difference between MT and ST_D_ was 1.35 ± 0.74 km/h. Slowing was least pronounced for the memory task and most pronounced for the reasoning task (significant ANOVA effect for Condition × Task), particularly when the latter was presented visually (significance for Condition × Modality, Task × Modality and Condition × Task × Modality). We further found a significant main effect for Group: older participants drove more slowly than young ones (*F* = 15.62, *p* = 0.00, $\eta_{p}^{2}$ = 0.11, df = 1, 122). The mean difference between young and older persons was 3.89 ± 0.41 km/h. We also observed significant main effects for Task (*F* = 78.98, *p* = 0.00, $\eta_{p}^{2}$ = 0.39, df = 1.92, 244) and for Modality (*F* = 22.25, *p* = 0.00, $\eta_{p}^{2}$ = 0.39, df = 1, 122): participants drove more slowly with the reasoning compared to the memory and the typing task, and they drove more slowly when tasks were presented visually rather than auditorily.

**Table 1:** ANOVA results for *mean velocity*.

Mean velocity	Condition	Group	Task	Modality	Condition × Group	Condition × Task	Condition × Modality	Group × Task	Group × Modality
*F*=	12.07	15.62	78.98	22.25	3.15	8.74	8.31	0.63	0.68
*p*=	**0.00^∗∗^**	**0.00^∗∗^**	**0.00^∗∗^**	**0.00^∗∗^**	0.08	**0.00^∗∗^**	**0.00^∗∗^**	0.53	0.41
$\eta_{p}^{2}$=	0.09	0.11	0.39	0.15	0.03	0.07	0.06	0.01	0.01
df=	1, 122	1, 122	1.92, 244	1, 122	1, 122	2, 244	1, 122	2, 121	1, 122

	**Task × Modality**	**Condition × Group × Task**	**Condition × Group × Modality**	**Condition × Task × Modality**	**Group × Task × Mod.**	**Condition × Group × Task × Modality**			

*F*=	22.14	1.09	2.53	22.25	0.96	2.25			
*p*=	**0.00^∗∗^**	0.34	0.11	**0.00^∗∗^**	0.38	0.11			
$\eta_{p}^{2}$=	0.15	0.01	0.02	0.15	0.01	0.01			
df=	2, 244	2, 121	1, 122	2, 244	2, 121	2, 121			

*^∗^p < 0.05, ^∗∗^p < 0.001.*

**FIGURE 6:**
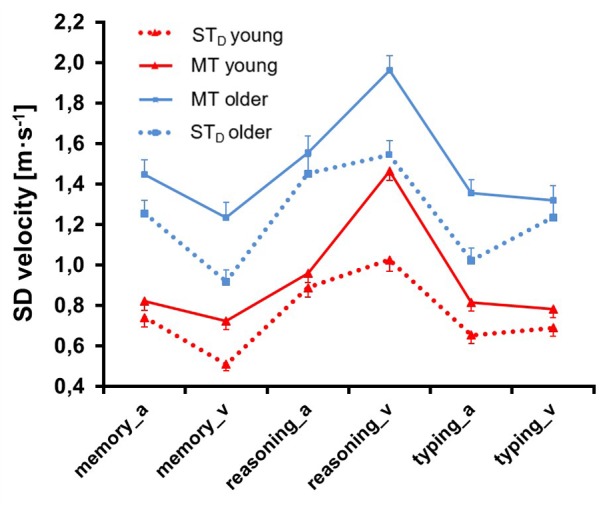
Standard deviation of velocity ± SE of both age groups in single task (ST_D_) and multitask (MT) conditions. Memory, reasoning and typing task were presented auditorily (_a) and visually (_v).

**Figure 6** illustrates corresponding data for the parameter *SD velocity*. ANOVA (see **Table 2**) revealed a significant main effect for Condition: speed variability scores were 0.75 ± 0.48 km/h higher in MT compared to ST_D_ (*F* = 32.60, *p* = 0.00, $\eta_{p}^{2}$ = 0.21, df = 1, 122). This increase was particularly pronounced for the visually presented reasoning task and when the typing task was presented auditorily (significance for Condition × Task, Modality, Condition × Modality, Task × Modality and Condition × Task × Modality). We further found a significant main effect for Group (*F* = 30.70, *p* = 0.00, $\eta_{p}^{2}$ = 0.20, df = 1, 122): variability scores were -1.87 ± 0.19 km/h higher in older compared to young persons. We also found a significant main effect for Task (*F* = 230.39, *p* = 0.00, $\eta_{p}^{2}$ = 0.65, df = 1.69, 206.19): variability scores were higher for the reasoning task compared to the memory and the typing task.

**Table 2:** ANOVA results for *SD velocity*.

SD velocity	Condition	Group	Task	Modality	Condition × Group	Condition × Task	Condition × Modality	Group × Task	Group × Modality
*F*=	32.60	30.70	230.39	4.67	0.32	5.51	23.08	0.49	0.97
*p*=	**0.00^∗∗^**	**0.00^∗∗^**	**0.00^∗∗^**	**0.03^∗^**	0.58	**0.01^∗^**	**0.00^∗∗^**	0.58	0.33
$\eta_{p}^{2}$=	0.21	0.20	0.65	0.04	0.00	0.04	0.16	0.00	0.01
df=	1, 122	1, 122	1.69, 206.19	1, 122	1, 122	1.84, 244	1, 122	1.69, 121	1, 122

	**Task × Modality**	**Condition × Group × Task**	**Condition × Group × Modality**	**Condition × Task × Modality**	**Group × Task × Modality**	**Condition × Group × Task × Modality**			

*F*=	80.79	0.86	2.57	47.96	1.29	0.11			
*p*=	**0.00^∗∗^**	0.42	0.11	**0.00^∗∗^**	0.28	0.88			
$\eta_{p}^{2}$=	0.40	0.01	0.02	0.28	0.01	0.00			
df=	2, 244	1.84, 121	1, 122	1.83, 223.52	1.93, 121	1.83, 121			

*^∗^p < 0.05, ^∗∗^p < 0.001.*

**FIGURE 7:**
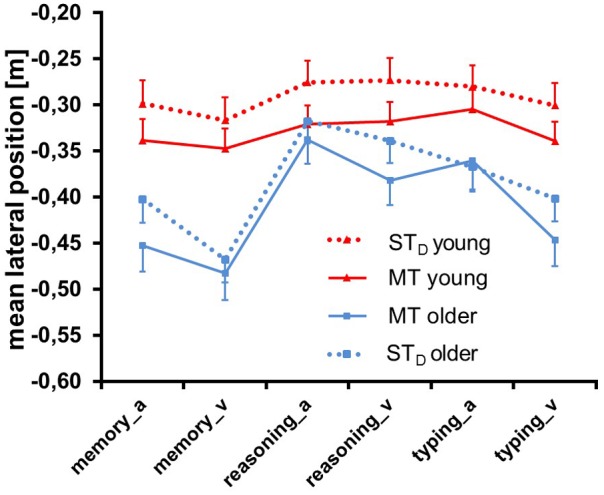
Mean lateral position ± SE of both age groups in single task (ST_D_) and multitask (MT) conditions. Memory, reasoning and typing task were presented auditorily (_a) and visually (_v).

**Figure 7** shows the parameter *mean lateral position* of both age groups in ST_D_ and in MT, separately for all six combinations of loading task and modality. ANOVA (see **Table 3**) yielded a significant main effect for Condition: participants drove more laterally in MT compared to ST_D_ (*F* = 11.10, *p* = 0.00, $\eta_{p}^{2}$ = 0.08, df = 1, 122). Mean difference between MT and ST_D_ was 0.12 ± 0.05 m. This shift toward the curb was larger when the memory task was presented visually and when the reasoning and typing tasks were presented auditorily, more so in older than in young persons [significance for Modality (*F* = 61.91, *p* = 0.00. $\eta_{p}^{2}$ = 0.34, df = 1, 122), Group × Modality, Task × Modality and Condition × Group × Modality]. The main effect for Group was not significant, but a significant effect of Task (*F* = 79.79, *p* = 0.00, $\eta_{p}^{2}$ = 0.40, df = 1.72, 209.82) and Group × Task emerged: participants drove more laterally when performing the memory task and this shift toward the curb was much more pronounced in older persons.

**Table 3:** ANOVA results for *mean lateral position*.

Mean lateral position	Condition	Group	Task	Modality	Condition × Group	Condition × Task	Condition × Modality	Group × Task	Group × Modality
*F*=	11.10	1.36	79.79	61.91	0.27	1.85	2.36	23.24	10.93
*p*=	**0.00^∗∗^**	0.25	**0.00^∗∗^**	**0.00^∗∗^**	0.61	0.16	0.13	**0.00^∗∗^**	**0.00^∗∗^**
$\eta_{p}^{2}$=	0.08	0.01	0.40	0.34	0.00	0.02	0.02	0.16	0.08
df =	1, 122	1, 122	1.72, 209.82	1, 122	1, 122	2, 244	1, 122	1, 121	1, 122

	**Task × Modality**	**Condition × Group × Task**	**Condition × Group × Modality**	**Condition × Task × Modality**	**Group × Task × Modality**	**Condition × Group × Task × Mod**			

*F*=	7.94	0.12	3.11	10.49	0.69	2.93			
*p*=	**0.00^∗∗^**	0.88	0.08	**0.00^∗∗^**	0.50	0.06			
$\eta_{p}^{2}$=	0.06	0.00	0.02	0.08	0.01	0.02			
df=	2, 244	1.95, 121	1, 122	2, 244	2, 121	2, 121			

*^∗∗^p < 0.001.*

**FIGURE 8:**
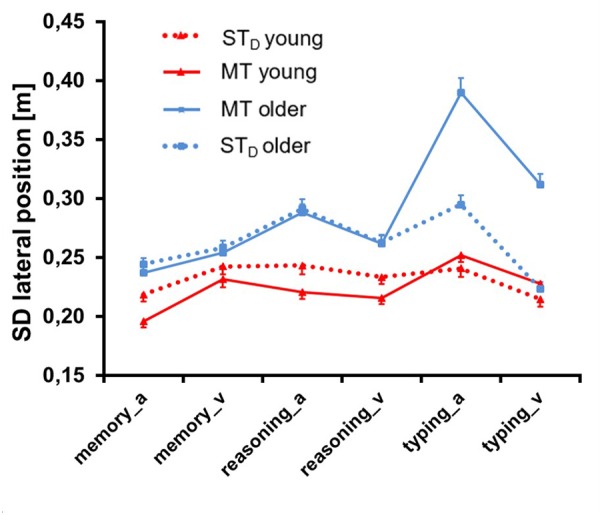
Standard deviation of lateral position ± SE of both age groups in single task (ST_D_) and multitask (MT) conditions. Memory, reasoning and typing task were presented auditorily (_a) and visually (_v).

**Figure 8** illustrates corresponding data for the parameter *SD lateral position*. ANOVA (see **Table 4**) revealed a significant main effect for Condition: scores were higher for MT compared to ST_D_ (*F* = 10.53, *p* = 0.00, $\eta_{p}^{2}$ = 0.08, df = 1, 122), but this was limited to older participants performing the typing task (significance for Task (*F* = 93.68, *p* = 0.00, $\eta_{p}^{2}$ = 0.43, df = 1.88, 244), Condition × Task, Condition × Group, Group × Task and Condition × Group × Task). Mean absolute difference between MT and ST was 0.03 ± 0.12 m. We also found a significant main effect for Group: scores were higher in older compared to young participants, with a mean difference of 0.17 ± 0.10 m (*F* = 20.82, *p* = 0.00, $\eta_{p}^{2}$ = 0.15, df = 1, 122). Finally, there was a significant main effect for Modality (*F* = 53.60, *p* = 0.00, $\eta_{p}^{2}$ = 0.31, df = 1, 122): scores were higher with auditory rather than visual task presentation, particularly for older participants in the typing task (significance for Group × Modality, Task × Modality and Group × Task × Modality).

**Table 4:** ANOVA results for *SD lateral position*.

SD lateral position	Condition	Group	Task	Modality	Condition × Group	Condition × Task	Condition × Modality	Group × Task	Group × Modality
*F*=	10.53	20.82	3.68	53.69	23.53	129.78	1.00	25.16	25.41
*p*=	**0.00^∗∗^**	**0.00^∗∗^**	**0.00^∗∗^**	**0.00^∗∗^**	**0.00^∗∗^**	**0.00^∗∗^**	0.32	**0.00^∗∗^**	**0.00^∗∗^**
$\eta_{p}^{2}$=	0.08	0.15	0.43	0.31	0.16	0.52	0.01	0.17	0.17
df=	1, 122	1, 122	1.88, 244	1, 122	1, 122	1.68, 204.55	1, 122	1.88, 121	1, 122

	**Task × Modality**	**Condtion × Group × Task**	**Condtion × Group × Modality**	**Condition × Task × Modality**	**Group × Task × Modality**	**Condition × Group × Task × Mod**			

*F*=	140.66	18.48	1.50	1.02	4.45	0.44			
*p*=	**0.00^∗∗^**	**0.00^∗∗^**	0.22	0.36	**0.01^∗^**	0.62			
$\eta_{p}^{2}$=	0.54	0.13	0.01	0.01	0.04	0.00			
df=	1.89, 230.33	1.68, 121	1, 122	1.82, 221.73	1.89, 121	1.82, 121			

*^∗^p < 0.05, ^∗∗^p < 0.001.*

We noticed that participants sometimes veered off their driving lane when they engaged in the typing task. 78% of older participants but only 40% of young ones reached the curb with their right wheels during at least one presentation of the typing task; this age difference is statistically significant (test of proportions: *p* < 0.001). Furthermore, 15% of older participants but 0% of young ones crossed the median with their left wheels at least once; this age difference is again statistically significant (*p* < 0.01).

### Loading Tasks

**FIGURE 9:**
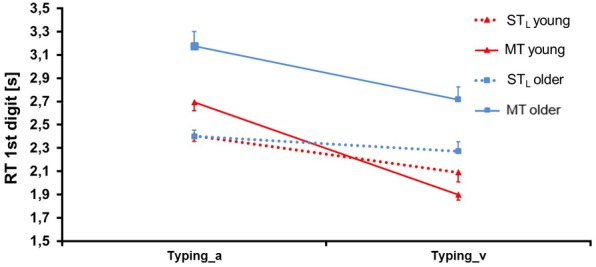
Reaction time (RT) ± SE in the typing task for both age groups in single task (ST_D_) and multitask (MT) conditions. The typing task was presented auditorily (_a) and visually (_v).

**Figure 9** depicts the RT in the typing task. ANOVA (see **Table 5**) yielded a significant main effect for Condition (*F* = 30.70, *p* = 0.00, $\eta_{p}^{2}$ = 0.20, df = 1, 122): RT was higher in MT compared to the ST_L_; however, this finding was limited to older participants (significance for Group × Condition). The mean difference between MT and ST_L_ was -0.32 ± 0.29 s. We further found a significant main effect for Group: RT of older participants was 0.37 ± 0.18 s higher than that of young ones (*F* = 10.22, *p* = 0.00, $\eta_{p}^{2}$ = 0.08, df = 1, 122). We also observed a significant main effect for Modality (*F* = 124.44, *p* = 0.00, $\eta_{p}^{2}$ = 0.50, df = 1, 122): RT was higher with auditory compared to visual presentation, more so in MT (significance for Condition × Modality) and in young persons (significance for Group × Modality).

**Table 5:** ANOVA results for *reaction time (RT)* of the typing task.

RT typing	Condition	Group	Modality	Condition × Group	Condition × Modality	Group × Modality	Condition × Group × Modality
*F*=	30.79	10.22	124.44	12.23	48.05	6.17	0.98
*p*=	**0.00^∗∗^**	**0.00^∗∗^**	**0.00^∗∗^**	**0.00^∗∗^**	**0.00^∗∗^**	**0.01^∗^**	0.32
$\eta_{p}^{2}$=	0.20	0.08	0.50	0.09	0.28	0.05	0.01
df=	1, 122	1, 122	1, 122	1, 122	1, 122	1, 122	1, 122

*^∗^p < 0.05, ^∗∗^p < 0.001.*

**FIGURE 10:**
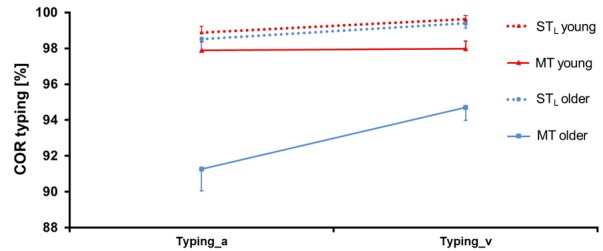
Correctness (COR) ± SE in the typing task for both age groups in single task (ST_D_) and multitask (MT) conditions. The typing task was presented auditorily (_a) and visually (_v).

**Figure 10** shows COR in the typing task. ANOVA (see **Table 6**) revealed a significant main effect for Condition (*F* = 66.00, *p* = 0.00, $\eta_{p}^{2}$ = 0.35, df = 1, 122): COR was lower by 0.036 ± 0.007 in MT compared to ST_L_, but this difference only occurred for older participants (significance for Condition × Group). There also was a significant main effect for Group (*F* = 8.56, *p* = 0.00, $\eta_{p}^{2}$ = 0.07, df = 1, 122) and for Modality (*F* = 8.78, *p* = 0.00, $\eta_{p}^{2}$ = 0.07, df = 1, 122), as COR was lower by 0.026 ± 0.012 in older compared to young persons, and lower for auditory compared to visual presentation.

**Table 6:** ANOVA results for *correctness (COR)* of the typing task.

COR typing	Condition	Group	Modality	Condition × Group	Condition × Modality	Group × Modality	Condition × Group × Modality
*F*=	66.00	8.56	8.78	10.36	1.41	0.11	0.04
*p*=	**0.00^∗∗^**	**0.00^∗∗^**	**0.00^∗∗^**	**0.00^∗∗^**	0.24	0.74	0.83
$\eta_{p}^{2}$=	0.35	0.07	0.07	0.08	0.01	0.00	0.00
df=	1, 122	1, 122	1, 122	1, 122	1, 122	1, 122	1, 122

*^∗∗^p < 0.001.*

**FIGURE 11:**
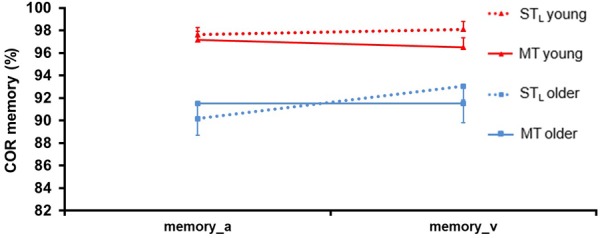
Correctness (COR) ± SE in the memory task for both age groups in single task (ST_D_) and multitask (MT) conditions. The memory task was presented auditorily (a) and visually (v).

Reaction time data from the memory task were not analyzed (see above), and COR data were not complete since the data sets of two older persons were lost for technical reasons. The remaining data are shown in **Figure 11**. ANOVA (see **Table 7**) revealed only a significant main effect for Group: COR was lower by 0.057 ± 0.011 in older compared to young participants (*F* = 19.31, *p* = 0.00, $\eta_{p}^{2}$ = 0.14, df = 1, 122).

**Table 7:** ANOVA results for *correctness (COR)* of the memory task.

COR memory	Condition	Group	Modality	Condition × Group	Condition × Modality	Group × Modality	Condition × Group × Modality
*F*=	0.74	19.31	0.78	0.04	2.34	0.78	0.53
*p* =	0.39	**0.00^∗∗^**	0.38	0.85	0.13	0.38	0.47
$\eta_{p}^{2}$=	0.01	0.14	0.01	0.00	0.02	0.01	0.00
df=	1, 122	1, 122	1, 122	1, 122	1, 122	1, 122	1, 122

*^∗∗^p < 0.001.*

## Discussion

This study deals with multitasking in simulated car driving. It differs from earlier work on this topic in two ways. First, we use not just one repetitive loading task but rather a mixed sequence of different loading tasks, to simulate the natural interplay of dual-tasking and task switching. Second, we compare driving performance of young to that of older persons. Our work addressed three hypotheses. According to one, performance of young and older persons will decrease under multitasking conditions. Indeed, we found significant main effects of Condition for all six outcome parameters. According to our second hypothesis, the effects of multitasking will be larger with visual compared to auditory loading tasks, because of structural interference. We found significance of Condition^∗^Modality for only three of our six parameters; we also observed three significant effects of Condition^∗^Task^∗^Modality, since effects of multitasking were sometimes smaller rather than larger with a visual loading task. We therefore found no unanimous support for the second hypothesis. Our third hypothesis stipulates that multitasking deficits may not be much larger in older compared to young persons, since cognitive decay is compensated by lifelong experience. Indeed, significance of Condition^∗^Group emerged for only one driving parameter and was qualified by significance of Condition^∗^Group^∗^Task: when multitasking, lateral lane variability increased in older persons more than in young ones, but only with the typing task. Accordingly, significance of Condition^∗^Group also emerged for both parameters related to typing. Our data therefore indicate that age-related deficits of multitasking emerge for some but not for other loading tasks, which adds partial support to our third hypothesis.

Compared to single-task driving, participants in MT drove at a lower speed, with a higher speed variability and at a more lateral lane position. Similarly, Chaparro et al. (2005), Horberry et al. (2006), Horrey and Wickens (2006), Strayer et al. (2006) reported lower speed and deficient lane keeping under dual- compared to single-task driving. As an example, Strayer et al. (2006) found driving speed to decrease by about 2.2 km/h when participants were talking on a mobile phone, while the decrease was about 1.4 km/h in the present multitasking study. More research is needed to find out whether our loading tasks were less disruptive than the task of Strayer et al. (2006) or, alternatively, whether multiple loading tasks are less disruptive than one single loading task. The observed reduction of driving speed and the more lateral lane position could represent compensatory strategies, implemented to avoid collisions with the leading car and with oncoming traffic in high-demand driving situations. The observed increase of speed variability could be a more direct marker of high demand: possibly, participants slowed down when their attention was focused on the loading task, and sped up to catch up with the leading car when attention was redirected to the driving task.

We further found that compared to young participants, older ones drove at a lower speed, with a higher speed variability and at a more lateral lane position. In other words, old age and multitasking had similar effects on driving, and possibly so for similar reasons, namely, a higher cognitive demand of driving. We also observed that older persons’ performance on the memory task was poorer than that of young ones, which concurs with the known age-related deficits of working memory (Salthouse and Babcock, 1991; Waters and Caplan, 2001; Voelcker-Rehage et al., 2006).

Chaparro et al. (2005) reported that a loading task had stronger effects on driving when it was presented visually rather than auditorily. We can’t confirm this observation unanimously, and therefore can’t claim unequivocal support for the structural-interference model (Gopher and Donchin, 1986; Duncan et al., 1997).

Although we hypothesized that age related deficits of multitasking are compensated by experience (see section “Introduction”) differential effects of age on multitasking were observed. Performance of older persons suffered more than that of young ones with the loading task ‘typing,’ not with ‘reasoning’ or ‘memory.’ Critically, this often let especially older persons veer off the lane when typing. The detrimental effect ‘typing’ on older persons could reflect the known age-related problems of attention engagement/disengagement (D’Aloisio and Klein, 1990), gaze control (Maltz and Shinar, 1999; Bock et al., 2015) and/or limb coordination (Darling et al., 1989; Ketcham et al., 2002). Since the keypad was located near the steering wheel, participants had to shift their attention, gaze and arm toward a new location in task ‘type,’ but not in the other two loading tasks. In any case, our finding could be of substantial relevance for the driving safety of older persons since activities similar to task ‘type’ are quite common in driving: drivers often operate radios, navigation systems and other dashboard instruments, open and close windows, adjust side and rear mirrors, and on longer trips may even reach for drinks and food located elsewhere in the car cabin. It would be interesting to know whether multitasking skills can be improved by practice. Previous work has shown that dual- but not single-task training improves dual-task performance (Silsupadol et al., 2009) and accordingly, multitask- but not dual-or single-task training may improve performance on a realistic multitask.

Future research should determine whether the effects of multitasking in our study, and their modulation by age, are similar, larger or smaller than those documented by traditional dual-task studies which disregarded the natural interplay of dual-tasking and task switching (see section “Introduction”). Furthermore, our present multitasking paradigm should be expanded to allow for more than two tasks at a given time; for example, participants could drive a car, memorize events in the environments and keep up a conversation all at the same time, then switch to driving, memorizing and typing, etc.

## Data Availability

The raw data supporting the conclusions of this manuscript will be made available by the authors, without undue reservation, to any qualified researcher.

## Author Contributions

CV-R and OB contributed conception and design of the study. KW wrote the first draft of the manuscript. CV-R, CJ, MH, UD, OB, and KW wrote sections of the manuscript. All authors contributed to manuscript revision and read and approved the submitted version.

## Conflict of Interest Statement

The authors declare that the research was conducted in the absence of any commercial or financial relationships that could be construed as a potential conflict of interest.
